# Altered vasoreactivity in neonatal rats with pulmonary hypertension associated with bronchopulmonary dysplasia: Implication of both eNOS phosphorylation and calcium signaling

**DOI:** 10.1371/journal.pone.0173044

**Published:** 2017-02-24

**Authors:** Eric Dumas de la Roque, Gwladys Smeralda, Jean-François Quignard, Véronique Freund-Michel, Arnaud Courtois, Roger Marthan, Bernard Muller, Christelle Guibert, Mathilde Dubois

**Affiliations:** 1 Univ. Bordeaux, Centre de recherche Cardio-Thoracique de Bordeaux, U1045, Bordeaux, France; 2 INSERM, Centre de recherche Cardio-Thoracique de Bordeaux, U1045, Bordeaux, France; 3 CHU de Bordeaux, Services de Réanimation Néonatale et Exploration Fonctionnelle Respiratoire, Centre d’Investigation Clinique (CIC 0005), Bordeaux, France; Augusta University, UNITED STATES

## Abstract

Bronchopulmonary dysplasia (BPD) consists of an arrest of pulmonary vascular and alveolar growth, with persistent hypoplasia of the pulmonary microvasculature and alveolar simplification. In 25 to 40% of the cases, BPD is complicated by pulmonary hypertension (BPD-PH) that significantly increases the risk of morbidity. *In vivo* studies suggest that increased pulmonary vascular tone could contribute to late PH in BPD. Nevertheless, an alteration in vasoreactivity as well as the mechanisms involved remain to be confirmed. The purpose of this study was thus to assess changes in pulmonary vascular reactivity in a murine model of BPD-PH. Newborn Wistar rats were exposed to either room air (normoxia) or 90% O_2_ (hyperoxia) for 14 days. Exposure to hyperoxia induced the well-known features of BPD-PH such as elevated right ventricular systolic pressure, right ventricular hypertrophy, pulmonary vascular remodeling and decreased pulmonary vascular density. Intrapulmonary arteries from hyperoxic pups showed decreased endothelium-dependent relaxation to acetylcholine without any alteration of relaxation to the NO-donor sodium nitroprusside. This functional alteration was associated with a decrease of lung eNOS phosphorylation at the Ser1177 activating site. In pups exposed to hyperoxia, serotonin and phenylephrine induced exacerbated contractile responses of intrapulmonary arteries as well as intracellular calcium response in pulmonary arterial smooth muscle cells (PASMC). Moreover, the amplitude of the store-operated Ca^2+^ entry (SOCE), induced by store depletion using a SERCA inhibitor, was significantly greater in PASMC from hyperoxic pups. Altogether, hyperoxia-induced BPD-PH alters the pulmonary arterial reactivity, with effects on both endothelial and smooth muscle functions. Reduced activating eNOS phosphorylation and enhanced Ca^2+^ signaling likely account for alterations of pulmonary arterial reactivity.

## Introduction

Preterm birth causes infants to be born while the lung is still immature and unable to maintain adequate gas exchange [1]. Consequently, preterm infants often require respiratory support including assisted ventilation and supplemental oxygen therapy. However, these life-saving interventions can induce complications, among which bronchopulmonary dysplasia (BPD) is one of the most serious. BPD consists more prominently of an arrest of pulmonary vascular and alveolar growth, with persistent hypoplasia of the pulmonary microvasculature and alveolar simplification [2]. A dysmorphic growth and an impaired function of the pulmonary vasculature can result in pulmonary hypertension (PH) and subsequently lead to right ventricular hypertrophy, in 25 to 40% of the cases of BPD [2,3]. PH severely increases morbidity of BPD and, since the underlying pathophysiology is poorly understood, there is no definitive treatment to prevent BPD associated PH (BPD-PH).

Pulmonary vascular histopathology anomalies are observed in BPD-PH. Disruption of vascular growth results in decreased vessel density throughout the pulmonary micro-capillary network [4–6]. Moreover, intimal hyperplasia and increased muscularization of small pulmonary arteries have also been described [6–8]. Although structural remodeling and dysmorphic vascular growth can contribute to PH in patients with BPD, PH in children with BPD remains commonly responsive to a combined treatment with oxygen and inhaled nitric oxide (NO) which then decreases pulmonary artery pressures to near-normal levels [9], thus suggesting that the increased pulmonary vascular tone also significantly contributes to late PH. Nevertheless, alterations of vasoreactivity remain to be confirmed and underlying mechanisms to be characterized.

Interestingly, animal studies demonstrated that an increase in pulmonary vascular pressure itself could induce impaired vascular growth and alveolarization in the developing lung, suggesting that hemodynamic stress may be an additional mechanism for abnormal lung structure in BPD. Indeed, in a fetal ovine model, chronically elevated pulmonary vascular pressure induced by surgical partial constriction of the ductus arteriosus led to morphological pulmonary alteration characteristic of BPD, such as increased pulmonary arteriolar wall thickness, reduced small pulmonary vascular density and reduced alveolarization [10]. Furthermore, reducing pulmonary vascular pressure with inhaled NO attenuated these lung injuries in a rat model of BPD [11]. Since alterations in vascular reactivity are involved in the increase in pulmonary vascular pressure and since the latter contributes to structural abnormalities of the lung observed in BPD, vasoreactivity thus appears as an important element to take into consideration in BPD. Pulmonary vascular tone is controlled by the variations in the cytosolic calcium concentration ([Ca^2+^]_cyt_) in pulmonary arterial smooth muscle cells (PASMC) [12,13] and by the release of vasorelaxant factors, such as NO by endothelial cells. Then, endothelial NO-synthase (eNOS) is a key determinant of perinatal pulmonary vascular tone, whose activity is regulated by phosphorylation, interaction with substrate and co-factors, shear stress and numerous agonists [14,15].

In this study, we used a previously described hyperoxic murine model to mimic BPD-PH [16]. Although lung signaling pathways involved in BPD-PH pathogenesis have been extensively studied in this model, there is little information on vasoreactivity. To assess pulmonary vascular reactivity, we evaluated *ex vivo* contractile and relaxant responses of intrapulmonary arteries isolated from control vs hyperoxic pups. We also studied cytosolic calcium responses to vasocontractile agents as well as both expression and phosphorylation of eNOS in the BPD-PH model. We found that hyperoxia induces endothelial dysfunction and increased vasoconstriction in pulmonary arteries. These functional alterations were associated with a decreased eNOS phosphorylation in the lung and with an increased [Ca^2+^]_cyt_ in PASMC.

## Materials and methods

### Animals

All animal studies conformed to the Guide for the Care and Use of Laboratory Animals (NIH Publication No. 85–23, revised 1996). Agreement (number A33-063-907) was obtained from the French authorities and all the protocols used were approved by the local ethics committee (“Comité d'éthique régional d'Aquitaine”, protocol number 2015091112352424APAFIS#1739). Aged-matched Wistar rat litters (Elevage Janvier, Le Genest St Isle, France) were exposed within 24 hours of birth either to room air (normoxia) or to 90% O_2_ (hyperoxia) in a plexiglass chamber with continuous O_2_ monitoring. Dams were rotated daily between normoxic and hyperoxic chambers to prevent maternal O_2_ toxicity. Pups were maintained in the chamber for 14 days and were analyzed at postnatal day 14. During this 14-day period, if the animals showed any signs of illness or distress, they were immediately euthanized with an intraperitoneal injection of pentobarbital (0.3 mg / g body weight). Exposure to hyperoxia resulted in a survival rate of 82.6%.

### Right ventricular systolic pressure and hypertrophy

Hemodynamic measurements were performed in nonventilated pups under isoflurane anesthesia (1.5%—2.5%, 2 L air / min). After thoracotomy, a heparin-filled hypodermic needle connected to a polyethylene catheter was placed into the right ventricular cavity by direct puncture of the right ventricle, as previously described [17]. The right ventricular systolic pressure was measured by using a fluid-filled force transducer (Baxter Uniflow gauge pressure transducer, Guyancourt, France) connected to the PowerLab recording unit (AD Instruments, Colorado Springs, CO) and analyzed with the LabChart software (AD Instruments). The whole procedure lasted less than 5 min for each animal. After completion of the measurement, animals were euthanized and their hearts and lungs were removed en bloc. The weight ratio or Fulton’s index: right ventricle / (left ventricle + septum) was calculated to assess right ventricular hypertrophy.

### Morphometric analysis of pulmonary arterioles

Pulmonary vascular remodeling was assessed by measuring the percentage of wall thickness of the arterioles as described previously [17]. Briefly, formaldehyde-fixed, paraffin-embedded, 4 μm thick left lung sections were stained with hematoxylin and eosin. All the arteries with external diameters below 100 μm on each lung section from 6 normoxic animals and 4 hyperoxic animals were considered; this corresponds to 35 to 43 vessels per lung section for normoxic animals and 12 to 32 vessels per lung section for hyperoxic animals. Analysis was performed in a blinded fashion by using the Image J software. The percentage of wall thickness was calculated as [(external wall areas)–(internal wall areas)] x 100 / external wall areas.

### Vessel density

Vessel density was evaluated by immunostaining of lung sections with anti-von Willebrand factor (vWF; Millipore, Temacula, CA) to visualize even the small non-muscularized vessels. Images were captured using NanoZoomer 2.0HT at 20X magnification. For vessel counting, 4 well inflated randomly acquired fields (3.5 mm^2^) were imaged per animal. The number of vessels (<100 μm) per field were counted and averaged per animal in a blinded fashion.

### Wire myograph studies

Intrapulmonary arteries (first order) were dissected and segments of 1.8–2.0 mm length were mounted in a Mulvany myograph (Danish Myo Technology, Aarhus, Denmark). Arteries were bathed in Krebs buffer (119 mM NaCl, 4.7 mM KCl, 1.5 mM CaCl_2_, 1.17 mM MgSO_4_, 1.18 mM KH_2_PO_4_, 25 mM NaHCO_3_ and 5.5 mM glucose) and maintained at 37°C. Arteries from control and hyperoxic pups were studied under a resting tension corresponding to equivalent transmural pressures of 30 and 60 mmHg, respectively, which were previously demonstrated to be the optimal resting tension in a passive length-tension relationship. The arteries functionality was checked by adding 80 mM KCl and no difference was observed between normoxic and hyperoxic arteries. The intrapulmonary artery contraction was evaluated with serotonin and phenylephrine. The relaxant responses to acetylcholine, sodium nitroprusside (SNP) and isoprenaline were determined after precontraction with phenylephrine (3.10^−7^ M, which corresponds approximately to 90% of the maximal contraction).

### Expression and phosphorylation of eNOS

For Western-blotting analysis of phosphorylated and nonphosphorylated eNOS, right lungs were quick-frozen in liquid nitrogen to preserve phosphorylations and subsequently homogenized in an ice-cold lysis buffer (50 mM Tris, pH 7.5, 150 mM NaCl, 0.1% SDS, 0.5% deoxycholate, 1% Triton X100) containing protease inhibitors (Sigma-Aldrich cocktail [Saint-Quentin Fallavier, France], 1 mM phenylmethylsulfonyl fluoride, 5 mM EDTA, 1 mM EGTA) and phosphatase inhibitors (10 mM Na fluoride, 2 mM Na orthovanadate and 500 nM calyculine A). Homogenates (30 μg of proteins) were separated by electrophoresis on SDS-PAGE and transferred to PVDF membrane. After blocking, membranes were incubated with antibodies against phospho-eNOS Ser1177 (Cell Signaling Technology, Danvers, MA), eNOS (BD Transduction Laboratories, Le Pont de Claix, France) or β-actin (Sigma-Aldrich) and anti-mouse or rabbit IgG peroxidase conjugated antibody (Thermo Scientific, Illkirch, France). Protein bands were visualized by chemiluminescence and quantified by using the Image Lab^™^ software (Biorad, Hercules, CA). Results were expressed as a signal ratio (phospho-eNOS to total eNOS or eNOS to β-actin).

### PASMC isolation and microspectrofluorimetric measurement of cytosolic calcium

PASMC were isolated from first order intrapulmonary artery as previously described [18]. Arteries from 3 different animals were simultaneously dissociated for each experiment and PASMC were plated on 25 mm coverslips and placed in a recording chamber on the stage of an inverted Nikon Eclipse/TE 200 microscope. The Ca^2+^-sensitive fluorescent probe indo-1 was used to record changes in [Ca^2+^]_cyt_ in cells [18]. PASMC were incubated at room temperature during 30 min in a physiological solution (130 mM NaCl, 5.6 mM KCl, 2 mM CaCl_2_, 1 mM MgCl_2_, 8 mM HEPES and 11 mM glucose) containing 5 μM of indo-1 penta-acetoxymethyl ester (indo1/AM; I-1223, Molecular Probes, Eugene, OR). Cells were then washed for 15 min in an indo-1/AM free solution and maintained at room temperature in the same solution before conducting the fluorescence measurements. Indo-1 was excited at 355 nm and the emitted fluorescence was recorded at 405 nm and 480 nm. The fluorescence ratio (λ_405_/λ_480_) was calculated and recorded online by using the pCLAMP software (pCLAMP 10 system, Molecular Devices, Foster City, CA). Δ ratio λ_405_/λ_480_ is the difference between the basal ratio and the ratio at maximal peak amplitude. Cells were constantly superfused with physiological solution at a rate of 3 mL / min. Serotonin (10 μM) or phenylephrine (10 μM) was delivered during 20 seconds by a pressure ejection from a pipette located close to the recorded cell. In order to study the store-operated Ca^2+^ entry (SOCE), PASMC were preincubated with thapsigargin (1 μM) for 15 min in a 0 mM Ca^2+^ solution prior to stimulation with a 2 mM Ca^2+^ solution.

### Data analysis

Data were expressed as mean ± SEM of n cells for calcium measurements, n vessels for wire myograph studies or n rats for all other studies. Statistical evaluation was performed by the non-parametric Mann–Whitney test for the right ventricular systolic pressure and hypertrophy, the vessel morphometry and density, the protein expression and phosphorylation as well as calcium measurements. The concentration-response curves were compared using analysis of variance. Values were considered statistically significant when P < 0.05.

## Results

### Characterization of the BPD-PH model

**Fig 1:**
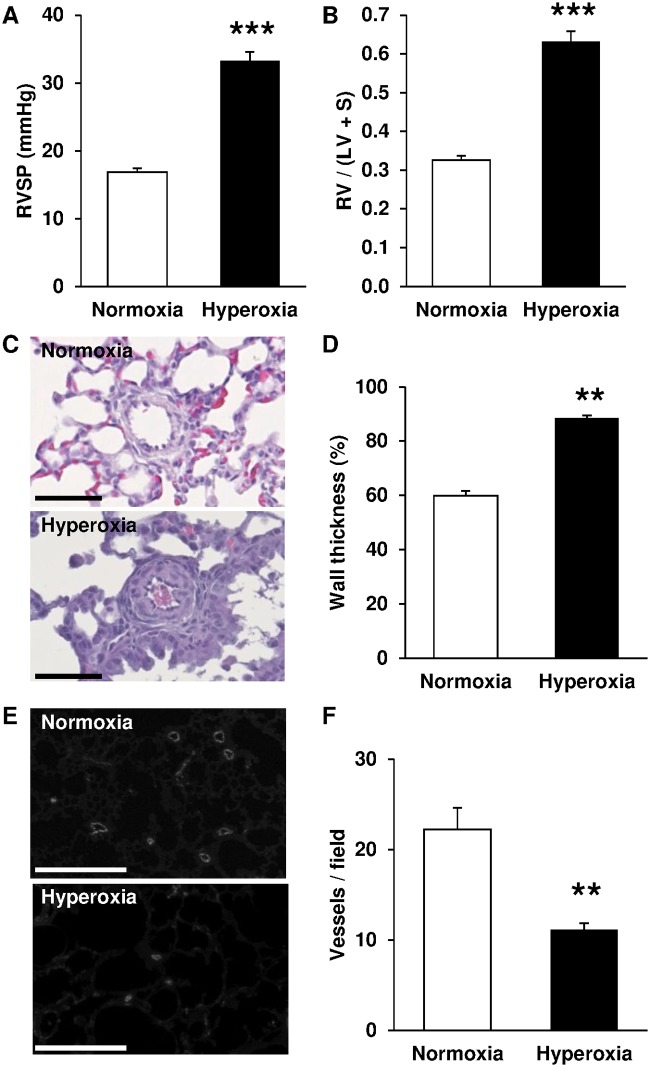
Exposure to hyperoxia induces features of BPD-PH in newborn rats. (A) Right ventricle systolic pressure (RSVP) was significantly increased by hyperoxia (*n* = 18) compared with normoxia (*n* = 9). (B) Fulton’s index (RV / (LV + S)) was significantly increased by hyperoxia (*n* = 24) compared with normoxia (*n* = 23). (C) Representative hematoxylin and eosin stained lung sections (scale bar = 50 μm). (D) Arterial wall thickness was significantly increased by hyperoxia (*n* = 4) compared with normoxia (*n* = 6). (E) Representative immunostaining of vWF in lung sections (scale bar = 250 μm). (F) Vessel density was significantly decreased by hyperoxia (n = 9) compared with normoxia (n = 9). ** *P* < 0.01; *** *P* < 0.001.

Right ventricular systolic pressure was significantly increased in pups exposed to hyperoxia for 14 days (Fig 1A). Hearts from those pups also shown a right ventricular hypertrophy, as evidenced by a significant increase in the weight ratio right ventricle / (left ventricle + septum) (Fig 1B). Hyperoxia also induced vascular remodeling by expansion of medial wall thickness in small pulmonary arteries (Fig 1C and 1D). Immunostaining of vWF showed that neonatal hyperoxia significantly decreased pulmonary vascular density (Fig 1E and 1F). All these modifications have previously been reported as features of BPD [19].

### Hyperoxia-induced alteration of vasomotor responses

**Fig 2:**
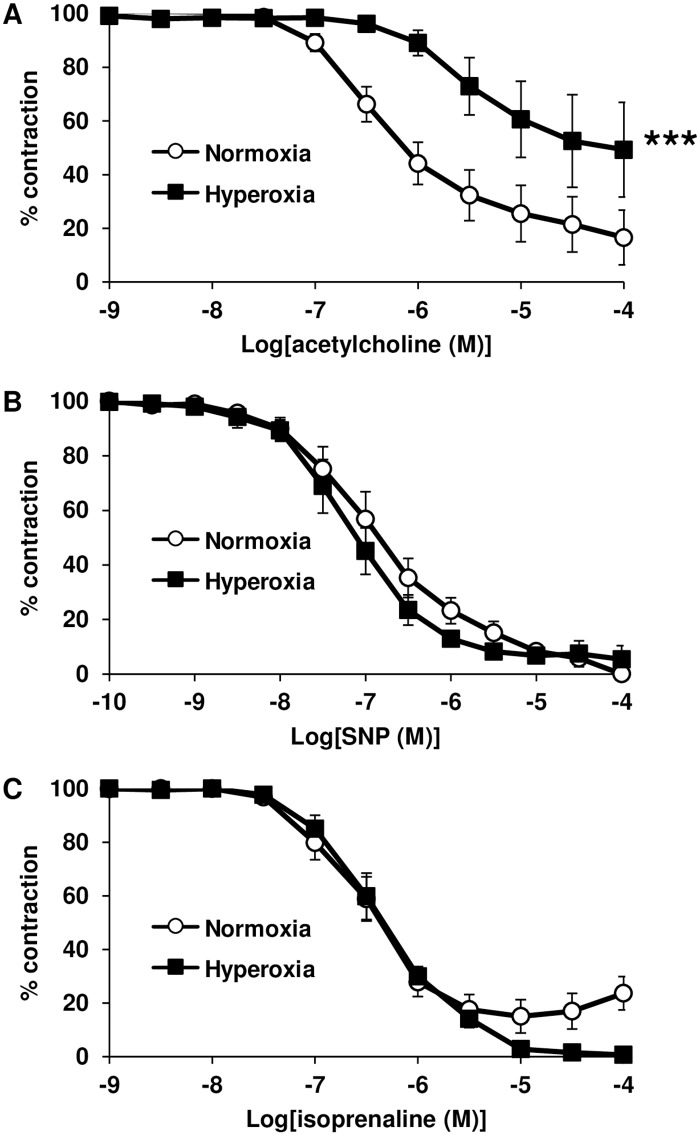
Chronic hyperoxia decreases relaxant response to acetylcholine in intrapulmonary arteries of newborn rats. (A) Arterial relaxant response to acetylcholine was significantly decreased by hyperoxia (*n* = 8 vessels from 3 animals) compared with normoxia (*n* = 15 vessels from 5 animals). (B) Arterial relaxant response to SNP was unaltered by hyperoxia (*n* = 9 vessels from 3 animals) compared with normoxia (*n* = 7 vessels from 3 animals). (C) Arterial relaxant response to isoprenaline was unaltered by hyperoxia (*n* = 8 vessels from 3 animals) compared with normoxia (*n* = 15 vessels from 5 animals). *** *P* < 0.001.

Endothelium-dependent relaxation to acetylcholine was significantly decreased in intrapulmonary arteries from pups exposed to hyperoxia, compared with normoxic controls (Fig 2A). However, relaxation to the NO-donor SNP was not altered following chronic hyperoxia (Fig 2B). This suggests that the decreased response to acetylcholine induced by hyperoxia is due to endothelial dysfunction. In this connection, relaxant response to the β_2_-adrenergic receptor agonist isoprenaline, which is NO- and endothelium-independent in rat intrapulmonary arteries [20] was not influenced by hyperoxia exposure (Fig 2C).

**Fig 3:**
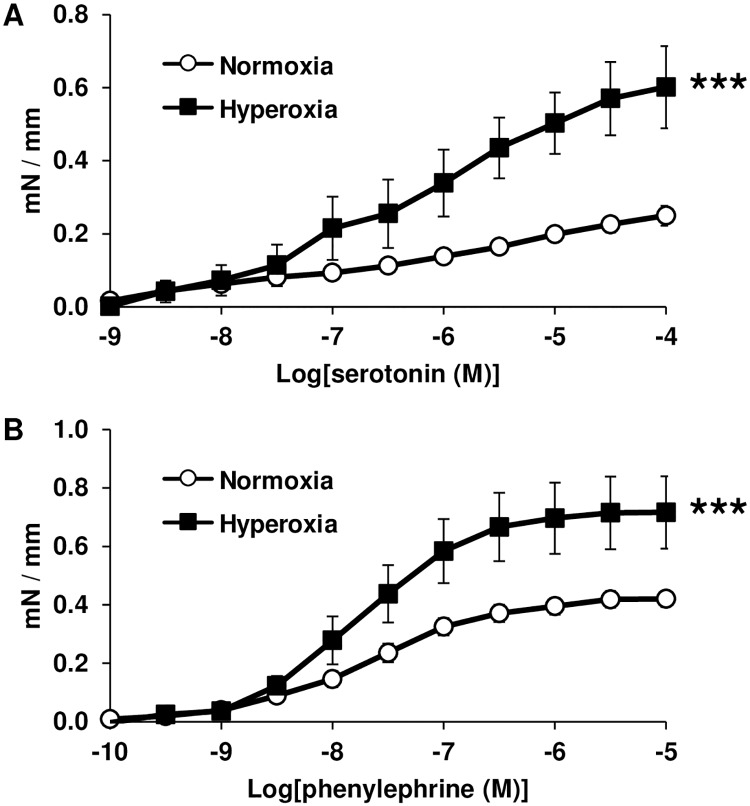
Chronic hyperoxia increases contractile response in intrapulmonary arteries of newborn rats. (A) Arterial contractile response to serotonin was significantly increased by hyperoxia (*n* = 8 vessels from 3 animals) compared with normoxia (*n* = 20 vessels from 6 animals). (B) Arterial contractile response to phenylephrine was significantly increased by hyperoxia (*n* = 11 vessels from 4 animals) compared with normoxia (*n* = 20 vessels from 6 animals). *** *P* < 0.001.

In addition, serotonin and phenylephrine-induced contraction were both significantly exacerbated in intrapulmonary arteries from pups exposed to hyperoxia, compared with normoxic controls (Fig 3).

### Hyperoxia-induced alteration of eNOS expression and phosphorylation

**Fig 4:**
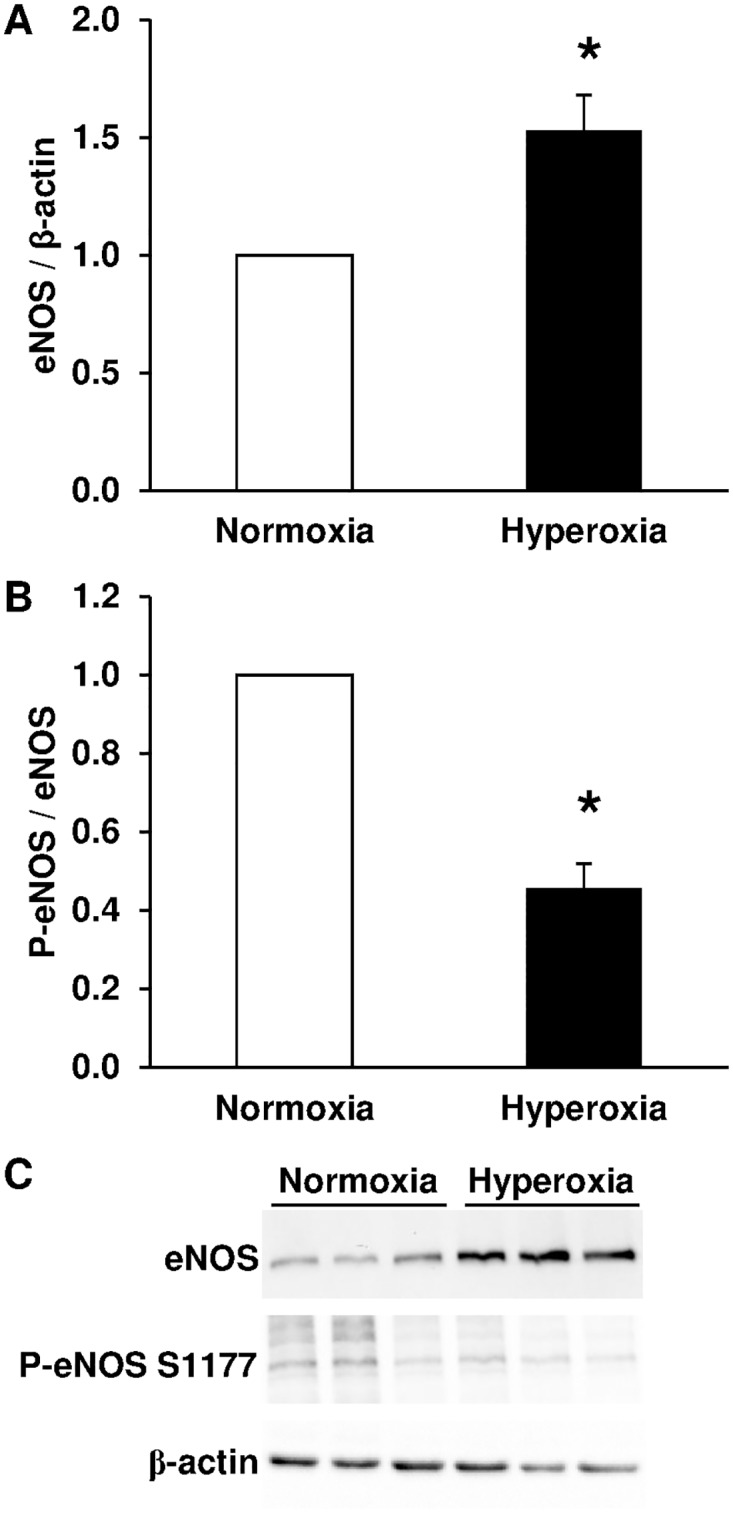
Chronic hyperoxia increases eNOS total expression but decreases eNOS phosphorylation in lungs from newborn rats. (A) eNOS total expression was significantly increased by hyperoxia (*n* = 8) compared with normoxia (*n* = 8). (B) eNOS phosphorylation at Ser1177 was significantly decreased by hyperoxia (*n* = 8) compared with normoxia (*n* = 8). (C) Representative Western-blots for total eNOS, eNOS phosphorylation at Ser1177 and β-actin. * *P* < 0.05.

Since NO-dependent relaxation of pulmonary arteries was decreased after exposure to hyperoxia, we investigated eNOS expression and phosphorylation. We found that chronic neonatal hyperoxia up-regulated eNOS expression in the lungs (Fig 4A and 4C), but reduced the proportion of eNOS phosphorylated at the Ser1177 activation site (Fig 4B and 4C).

### Hyperoxia-induced increase in basal [Ca^2+^]_cyt_ and Ca^2+^ response to serotonin and phenylephrine in PASMC

**Fig 5:**
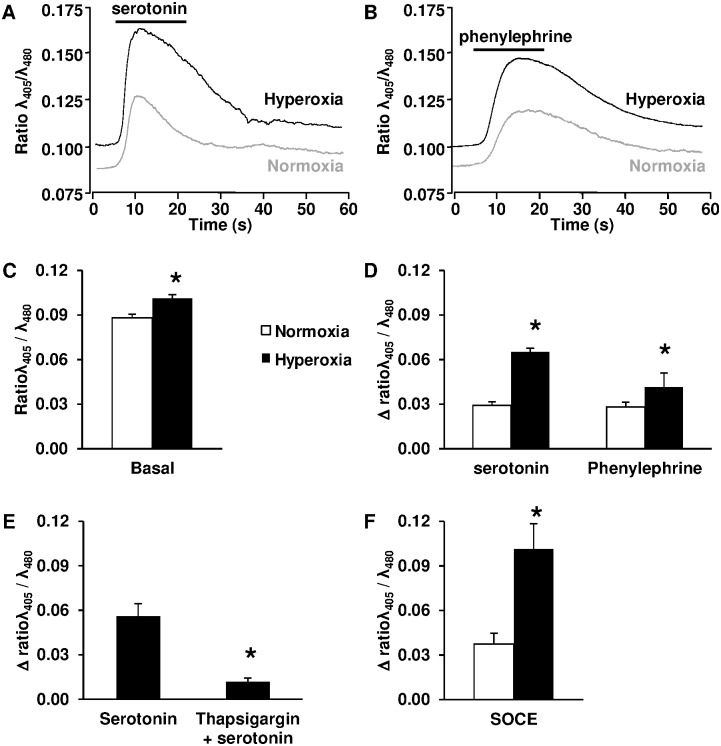
Chronic hyperoxia increases basal [Ca^2+^]_cyt_ and Ca^2+^ response in PASMC from newborn rats. (A) Representative Ca^2+^ response to serotonin in PASMC. (B) Representative Ca^2+^ response to phenylephrine in PASMC. (C) Basal [Ca^2+^]_cyt_ was significantly increased in PASMC from hyperoxic pups (*n* = 90) compared with normoxia (*n* = 90). (D) Ca^2+^ response to serotonin and phenylephrine were significantly increased in PASMC from hyperoxic pups (*n* = 35 and 20, respectively) compared with PASMC from normoxic pups (*n* = 33 and 20, respectively). (E) Ca^2+^ response to serotonin in PASMC from hyperoxic pups was significantly decreased by thapsigargin (*n* = 6). (F) SOCE was significantly increased in PASMC from hyperoxic pups (*n* = 11) compared with PASMC from normoxic pups (*n* = 12). * *P* < 0.05.

We investigated Ca^2+^ signaling in the context of increased vascular contractile response by hyperoxia. Basal [Ca^2+^]_cyt_ was slightly but significantly enhanced in PASMC isolated from pups exposed to hyperoxia, compared with normoxic controls (Fig 5A, 5B and 5C). Serotonin and phenylephrine both induced a rapid [Ca^2+^]_cyt_ increase in PASMC (Fig 5A and 5B). This Ca^2+^ response was strongly amplified in PASMC isolated from pups exposed to hyperoxia, compared with normoxic controls (Fig 5A, 5B and 5D). Serotonin-mediated [Ca^2+^]_cyt_ increase in PASMC is due to a Ca^2+^ release from reticulum store as it was reduced in the presence of thapsigargin, an inhibitor of the sarco(endo)plasmic reticulum Ca^2+^ ATPase (SERCA) (Fig 5E). Replenishment of reticulum by SOCE mechanism was greatly enhanced in PASMC from hyperoxic pups as compared with normoxic controls (Fig 5F).

## Discussion

Although altered vascular tone contributes to the pathophysiology of BPD-PH, vasoreactivity has been poorly examined in this disease. The aim of this study was thus to characterize the modification in contractile and relaxant pulmonary vascular responses and to determine underlying mechanisms. We demonstrate that, in a murine model of BPD-PH, intrapulmonary arterial reactivity is reduced in response to the endothelium-dependent relaxing-agent acetylcholine and exacerbated in response to contractile agents. Because acetylcholine induces vasorelaxation through stimulation of NO production by eNOS, the altered relaxant response to acetylcholine can be explained by the reduced eNOS phosphorylation at Ser1177, which decreases its activity. On the other hand, the enhanced contractility could be related to an increase in [Ca^2+^]_cyt_ in PASMC, that may be attributed to heightened SOCE activity and/or calcium release from intracellular calcium stores.

In this study, we used a previously described murine model to mimic BPD-PH [16]. Rodents are well suited for the study of BPD because their lungs at birth are in the saccular stage [16], structurally similar to human neonates born at 24 to 28 weeks of gestation. This model was reliable because pups exposed to hyperoxia consistently developed lung lesions, such as decreased vascular density and increased pulmonary vascular remodeling. These changes in lung structure closely mimic the histological features of the disorganized lung architecture observed in human infants suffering from BPD-PH [3,16,21]. These morphologic changes are accompanied by elevated right ventricular pressure which leads to right ventricular hypertrophy, a cardinal feature of PH.

We ascribed the altered vascular relaxation to eNOS activity although conflicting data exist regarding the effect of chronic hyperoxia on eNOS lung expression in the newborn rat. Such discrepancies could result from differences in the experimental design regarding percentage of O_2_, age of onset and duration of hyperoxia. While some authors report, in accordance with our results, an increase in eNOS lung protein content after exposure of pups to 60% O_2_ from day 0 to day 14 or 95% O_2_ from day 0 to day 8 or from day 3 to day 10 [22–24], others report that eNOS lung expression was either reduced after exposure of pups to 95% O_2_ from day 1 to day 7 [25] or unaltered after exposure of pups to 60% O_2_ from day 1 to day 14 [26]. Moreover, several studies demonstrated an alteration of the NO—soluble guanylate-cyclase (sGC)–cGMP signaling pathway in the hyperoxic newborn rat model. Physiologically, endothelial cells can release NO that stimulates sGC in PASMC to produce cGMP, which then induces vasodilatation. The 14-day exposure of pups to 75% O_2_ decreased sGC activity and lowered cGMP levels in small pulmonary arteries [6]. Exposure of pups to 95% O_2_ from day 5 to day 12 also reduced cGMP levels in the lungs [27]. Consistent with those observations, the 14-day exposure of pups to 60% O_2_ decreased NO release from pulmonary arteries [26]. These results are in accordance with our demonstration that, despite an increase in total eNOS expression, phosphorylation of eNOS at the Ser1177 activating site [15] is reduced by hyperoxia, suggesting that eNOS is less able to produce NO and to activate sGC. Furthermore, a decrease in eNOS activity is consistent with the altered relaxant response to acetylcholine we observed in pulmonary arteries from hyperoxic newborn rats. In the present study, endothelial dysfunction was demonstrated by altered relaxant response to acetylcholine without altered response to the NO-donor SNP in intrapulmonary arteries from hyperoxic pups. The unaltered relaxant capacity of smooth muscle was also confirmed in this model by an unchanged relaxant response to the β_2_-adrenergic receptor agonist isoprenaline, which is NO- and endothelium-independent in rat intrapulmonary arteries [20].

The additional role of NO in regulating normal lung maturation is supported by studies in which NO-donors increase branching morphogenesis of cultured fetal rat lungs [28]. Furthermore, other studies demonstrated that eNOS-deficient mouse pup lungs had decreased both microvascular development and expression of angiogenic factors, such as vascular endothelial growth factor-A, Flk-1, and TIE2 [29]. NO also seems to regulate the development of the injured newborn lung in various models. For example, eNOS-deficient mouse pups exposed to mild hypoxia show decreased pulmonary vessel density and alveolarization [30]. Furthermore, exposure to exogenous NO decreased abnormal PASMC proliferation in rat pups treated with monocrotaline, a toxic alkaloid that induces pulmonary hypertension [31], and improved pulmonary vessel density and alveolarization in rat pups exposed to hyperoxia [25]. These studies suggest a protective effect of NO on the injured newborn lung. Consequently, the alterations we observed in eNOS phosphorylation and endothelial NO-dependent relaxation may not only be implicated in vascular tone and PH but also in altered pulmonary development, although this hypothesis requires further investigation. Finally, since the NO-sGC-cGMP axis modulates vascular tone by regulating PASMC [Ca^2+^]_cyt_, the decrease in eNOS phosphorylation at Ser1177 could also contribute to the increase in basal [Ca^2+^]_cyt_ in PASMC isolated from pups exposed to hyperoxia as later discussed.

This study provides the first evidence that Ca^2+^ signaling is modified in PASMC from newborn rat with BPD-PH. Interestingly, Ca^2+^ signaling in PASMC plays a central role both in vasoconstriction, through its pivotal effect on PASMC contraction, and in vascular remodeling, through its stimulatory effect on PASMC proliferation and migration [32,33]. We demonstrated that the resting [Ca^2+^]_cyt_ is higher in the PASMC from hyperoxic newborn rats than in those from controls. Additionally, the amplitude of SOCE, induced by store depletion using thapsigargin, a SERCA inhibitor, was significantly greater in PASMC from pups with BPD-PH. Moreover, in PASMC from pups with BPD-PH, the Ca^2+^ response to serotonin is increased compared with control cells. SOCE, first described as capacitative Ca^2+^ entry, corresponds to the opening of store-operated Ca^2+^ channels (SOC) on the plasma membrane, due to depletion of Ca^2+^ from the endoplasmic reticulum. This process allows Ca^2+^ to fiow into the cytosol and Ca^2+^ is then sequestered into the endoplasmic reticulum by the SERCA, thus replenishing Ca^2+^ stores. As previously demonstrated in PASMC from adult rats [34], Ca^2+^ response to serotonin involves the release of Ca^2+^ from intracellular stores, since it is inhibited by thapsigargin. The enhanced SOCE activity induced by hyperoxia allows better replenishment of the reticulum, which may explain the increase in serotonin-mediated Ca^2+^ response, and consequently the increase in contractile response.

In accordance with our results, studies in fetal airway smooth muscle cells have also demonstrated that hyperoxia enhances fetal airway contractility via an increase in the amplitude of Ca^2+^ responses to histamine [35,36]. Furthermore, as in the current model, the resting [Ca^2+^]_cyt_ is higher in PASMC from adult patients with idiopathic pulmonary arterial hypertension or adult rats with hypoxic PH [37,38]. Similarly, the contraction to serotonin is also increased in adult rats with hypoxic PH [38]. Additionally, the amplitude of SOCE is increased in PASMC from adult patients with idiopathic pulmonary arterial hypertension compared with control cells [32,39]. When [Ca^2+^]_cyt_ increases, Ca^2+^ binds to calmodulin, which then activates myosin light chain kinase and consequently induces phosphorylation of contractile proteins and vessel contraction. Sustained pulmonary vasoconstriction is partly responsible for the elevated pulmonary vascular resistance and for the pulmonary arterial pressure observed in adult patients with idiopathic pulmonary arterial hypertension [32]. Likewise, we can hypothesize that, in our model of BPD-PH, the elevated [Ca^2+^]_cyt_ we observed contributes to elevated pulmonary arterial pressure, reflected by the increase in right ventricular systolic pressure.

Besides its promotion of contraction, the elevation of intracellular Ca^2+^, and especially SOCE, can activate signal transduction pathways important for the stimulation of transcription factors required for cell-cycle progression and proliferation, a key component of vascular remodeling [32]. In normal PASMC, the resting [Ca^2+^]_cyt_ as well as the increase in [Ca^2+^]_cyt_ due to SOCE are both greater in proliferating PASMC than in growth-arrested cells [40,41]. In animal models of adult PH as well as in human PASMC exposed to hypoxia, it has been demonstrated that increased PASMC proliferation is dependent on enhanced SOCE [42–44]. In the same line of evidence, treatments that reduce SOCE in these models also reduce PASMC proliferation. These results suggest that elevated [Ca^2+^]_cyt_ and intracellularly stored Ca^2+^ play an important role in PASMC growth, especially in the context of PH. In our model of BPD-PH, we report remodeling of pulmonary arteries with increased medial wall thickness, reflecting an increase in PASMC proliferation. Since we observed enhanced SOCE in PASMC, SOCE and proliferation in PASMC in BPD-PH may be closely related, although this assumption requires further investigation.

In conclusion, in the present study, we have shown that pulmonary vascular reactivity is altered in a model of murine BPD-PH with both endothelial and smooth muscle dysfunctions. The cellular mechanisms involve a decreased eNOS phosphorylation at its activating site and an increase in resting [Ca^2+^]_cyt_ and SOCE in PASMC. The present findings argue in favor of future studies to investigate the influence of decreased eNOS activity and Ca^2+^ signaling modification on altered lung maturation, including PASMC proliferation and migration in order to closely link vascular contractility and remodeling in this disease.

## References

[1] Smith LJ, McKay KO, van Asperen PP, Selvadurai H, Fitzgerald DA. Normal development of the lung and premature birth. Paediatr Respir Rev 2010;11: 135–142. 10.1016/j.prrv.2009.12.006 20692626

[2] Baker CD, Alvira CM. Disrupted lung development and bronchopulmonary dysplasia: opportunities for lung repair and regeneration. Curr Opin Pediatr 2014;26: 306–314. 10.1097/MOP.0000000000000095 24739494 PMC4121955

[3] Berkelhamer SK, Mestan KK, Steinhorn RH. Pulmonary hypertension in bronchopulmonary dysplasia. Semin Perinatol 2013;37: 124–131. 10.1053/j.semperi.2013.01.009 23582967 PMC4464837

[4] Bhatt AJ, Pryhuber GS, Huyck H, Watkins RH, Metlay LA, Maniscalco WM. Disrupted pulmonary vasculature and decreased vascular endothelial growth factor, Flt-1, and TIE-2 in human infants dying with bronchopulmonary dysplasia. Am J Respir Crit Care Med 2001;164: 1971–1980. 10.1164/ajrccm.164.10.2101140 11734454

[5] Coalson JJ. Pathology of new bronchopulmonary dysplasia. Semin Neonatol 2003;8: 73–81. 12667832 10.1016/s1084-2756(02)00193-8

[6] Lee KJ, Berkelhamer SK, Kim GA, Taylor JM, O'Shea KM, Steinhorn RH, et al Disrupted pulmonary artery cyclic guanosine monophosphate signaling in mice with hyperoxia-induced pulmonary hypertension. Am J Respir Cell Mol Biol 2014;50: 369–378. 10.1165/rcmb.2013-0118OC 24032519 PMC3930949

[7] Bush A, Busst CM, Knight WB, Hislop AA, Haworth SG, Shinebourne EA. Changes in pulmonary circulation in severe bronchopulmonary dysplasia. Arch Dis Child 1990;65: 739–745. 2117421 10.1136/adc.65.7.739 PMC1792458

[8] Gorenflo M, Vogel M, Obladen M. Pulmonary vascular changes in bronchopulmonary dysplasia: a clinicopathologic correlation in short- and long-term survivors. Pediatr Pathol 1991;11: 851–866. 1775401 10.3109/15513819109065482

[9] Mourani PM, Ivy DD, Gao D, Abman SH. Pulmonary vascular effects of inhaled nitric oxide and oxygen tension in bronchopulmonary dysplasia. Am J Respir Crit Care Med 2004;170: 1006–1013. 10.1164/rccm.200310-1483OC 15184202

[10] Grover TR, Parker TA, Balasubramaniam V, Markham NE, Abman SH. Pulmonary hypertension impairs alveolarization and reduces lung growth in the ovine fetus. Am J Physiol Lung Cell Mol Physiol 2005;288: L648–654. 10.1152/ajplung.00288.2004 15579625

[11] Tourneux P, Markham N, Seedorf G, Balasubramaniam V, Abman SH. Inhaled nitric oxide improves lung structure and pulmonary hypertension in a model of bleomycin-induced bronchopulmonary dysplasia in neonatal rats. Am J Physiol Lung Cell Mol Physiol 2009;297: L1103–1111. 10.1152/ajplung.00293.2009 19837849

[12] Berridge MJ. Smooth muscle cell calcium activation mechanisms. J Physiol 2008;586: 5047–5061. 10.1113/jphysiol.2008.160440 18787034 PMC2652144

[13] Wray S, Burdyga T. Sarcoplasmic reticulum function in smooth muscle. Physiol Rev 2010;90: 113–178. 10.1152/physrev.00018.2008 20086075

[14] Levy M, Maurey C, Dinh-Xuan AT, Vouhe P, Israel-Biet D. Developmental expression of vasoactive and growth factors in human lung. Role in pulmonary vascular resistance adaptation at birth. Pediatr Res 2005;57: 21R–25R. 10.1203/01.PDR.0000159575.58834.8D 15817500

[15] Fleming I. Molecular mechanisms underlying the activation of eNOS. Pflugers Arch 2010;459: 793–806. 10.1007/s00424-009-0767-7 20012875

[16] Hilgendorff A, Reiss I, Ehrhardt H, Eickelberg O, Alvira CM. Chronic lung disease in the preterm infant. Lessons learned from animal models. Am J Respir Cell Mol Biol 2014;50: 233–245. 10.1165/rcmb.2013-0014TR 24024524 PMC5455410

[17] Dubois M, Delannoy E, Duluc L, Closs E, Li H, Toussaint C, et al Biopterin metabolism and eNOS expression during hypoxic pulmonary hypertension in mice. PLoS One 2013;8: e82594 10.1371/journal.pone.0082594 24312428 PMC3842263

[18] Gilbert G, Ducret T, Marthan R, Savineau JP, Quignard JF. Stretch-induced Ca2+ signalling in vascular smooth muscle cells depends on Ca2+ store segregation. Cardiovasc Res 2014;103: 313–323. 10.1093/cvr/cvu069 24692174

[19] Thebaud B, Abman SH. Bronchopulmonary dysplasia: where have all the vessels gone? Roles of angiogenic growth factors in chronic lung disease. Am J Respir Crit Care Med 2007;175: 978–985. 10.1164/rccm.200611-1660PP 17272782 PMC2176086

[20] Pourageaud F, Leblais V, Bellance N, Marthan R, Muller B. Role of beta2-adrenoceptors (beta-AR), but not beta1-, beta3-AR and endothelial nitric oxide, in beta-AR-mediated relaxation of rat intrapulmonary artery. Naunyn Schmiedebergs Arch Pharmacol 2005;372: 14–23. 10.1007/s00210-005-1082-2 16133491

[21] Baker CD, Abman SH, Mourani PM. Pulmonary Hypertension in Preterm Infants with Bronchopulmonary Dysplasia. Pediatr Allergy Immunol Pulmonol 2014;27: 8–16. 10.1089/ped.2013.0323 24669351 PMC3961769

[22] Radomski A, Sawicki G, Olson DM, Radomski MW. The role of nitric oxide and metalloproteinases in the pathogenesis of hyperoxia-induced lung injury in newborn rats. Br J Pharmacol 1998;125: 1455–1462. 10.1038/sj.bjp.0702216 9884073 PMC1565728

[23] Potter CF, Kuo NT, Farver CF, McMahon JT, Chang CH, Agani FH, et al Effects of hyperoxia on nitric oxide synthase expression, nitric oxide activity, and lung injury in rat pups. Pediatr Res 1999;45: 8–13. 10.1203/00006450-199901000-00003 9890602

[24] Grisafi D, Tassone E, Dedja A, Oselladore B, Masola V, Guzzardo V, et al L-citrulline prevents alveolar and vascular derangement in a rat model of moderate hyperoxia-induced lung injury. Lung 2012;190: 419–430. 10.1007/s00408-012-9382-z 22430123

[25] Lin YJ, Markham NE, Balasubramaniam V, Tang JR, Maxey A, Kinsella JP, et al Inhaled nitric oxide enhances distal lung growth after exposure to hyperoxia in neonatal rats. Pediatr Res 2005;58: 22–29. 10.1203/01.PDR.0000163378.94837.3E 15879297

[26] Belik J, Jankov RP, Pan J, Yi M, Chaudhry I, Tanswell AK. Chronic O2 exposure in the newborn rat results in decreased pulmonary arterial nitric oxide release and altered smooth muscle response to isoprostane. J Appl Physiol (1985) 2004;96: 725–730. 14565964 10.1152/japplphysiol.00825.2003

[27] Sopi RB, Haxhiu MA, Martin RJ, Dreshaj IA, Kamath S, Zaidi SI. Disruption of NO-cGMP signaling by neonatal hyperoxia impairs relaxation of lung parenchyma. Am J Physiol Lung Cell Mol Physiol 2007;293: L1029–1036. 10.1152/ajplung.00182.2007 17660329

[28] Young SL, Evans K, Eu JP. Nitric oxide modulates branching morphogenesis in fetal rat lung explants. Am J Physiol Lung Cell Mol Physiol 2002;282: L379–385. 10.1152/ajplung.00462.2000 11839530

[29] Han RN, Babaei S, Robb M, Lee T, Ridsdale R, Ackerley C, et al Defective lung vascular development and fatal respiratory distress in endothelial NO synthase-deficient mice: a model of alveolar capillary dysplasia? Circ Res 2004;94: 1115–1123. 10.1161/01.RES.0000125624.85852.1E 15016731

[30] Balasubramaniam V, Tang JR, Maxey A, Plopper CG, Abman SH. Mild hypoxia impairs alveolarization in the endothelial nitric oxide synthase-deficient mouse. Am J Physiol Lung Cell Mol Physiol 2003;284: L964–971. 10.1152/ajplung.00421.2002 12588707

[31] Roberts JD Jr., Chiche JD, Weimann J, Steudel W, Zapol WM, Bloch KD. Nitric oxide inhalation decreases pulmonary artery remodeling in the injured lungs of rat pups. Circ Res 2000;87: 140–145. 10903998 10.1161/01.res.87.2.140

[32] Kuhr FK, Smith KA, Song MY, Levitan I, Yuan JX. New mechanisms of pulmonary arterial hypertension: role of Ca(2)(+) signaling. Am J Physiol Heart Circ Physiol 2012;302: H1546–1562. 10.1152/ajpheart.00944.2011 22245772 PMC3330808

[33] Wagenaar GT, Hiemstra PS, Gosens R. Therapeutic potential of soluble guanylate cyclase modulators in neonatal chronic lung disease. Am J Physiol Lung Cell Mol Physiol 2015;309: L1037–1040. 10.1152/ajplung.00333.2015 26432873

[34] Yuan XJ, Bright RT, Aldinger AM, Rubin LJ. Nitric oxide inhibits serotonin-induced calcium release in pulmonary artery smooth muscle cells. Am J Physiol 1997;272: L44–50. 9038901 10.1152/ajplung.1997.272.1.L44

[35] Britt RD Jr., Thompson MA, Kuipers I, Stewart A, Vogel ER, Thu J, et al Soluble guanylate cyclase modulators blunt hyperoxia effects on calcium responses of developing human airway smooth muscle. Am J Physiol Lung Cell Mol Physiol 2015;309: L537–542. 10.1152/ajplung.00232.2015 26254425 PMC4572415

[36] Thompson MA, Britt RD Jr., Kuipers I, Stewart A, Thu J, Pandya HC, et al cAMP-mediated secretion of brain-derived neurotrophic factor in developing airway smooth muscle. Biochim Biophys Acta 2015;1853: 2506–2514. 10.1016/j.bbamcr.2015.06.008 26112987 PMC4558218

[37] Yu Y, Keller SH, Remillard CV, Safrina O, Nicholson A, Zhang SL, et al A functional single-nucleotide polymorphism in the TRPC6 gene promoter associated with idiopathic pulmonary arterial hypertension. Circulation 2009;119: 2313–2322. 10.1161/CIRCULATIONAHA.108.782458 19380626 PMC2749566

[38] Rodat L, Savineau JP, Marthan R, Guibert C. Effect of chronic hypoxia on voltage-independent calcium influx activated by 5-HT in rat intrapulmonary arteries. Pflugers Arch 2007;454: 41–51. 10.1007/s00424-006-0178-y 17146678

[39] Song MY, Makino A, Yuan JX. STIM2 Contributes to Enhanced Store-operated Ca Entry in Pulmonary Artery Smooth Muscle Cells from Patients with Idiopathic Pulmonary Arterial Hypertension. Pulm Circ 2011;1: 84–94. 10.4103/2045-8932.78106 21709766 PMC3121304

[40] Golovina VA, Platoshyn O, Bailey CL, Wang J, Limsuwan A, Sweeney M, et al Upregulated TRP and enhanced capacitative Ca(2+) entry in human pulmonary artery myocytes during proliferation. Am J Physiol Heart Circ Physiol 2001;280: H746–755. 11158974 10.1152/ajpheart.2001.280.2.H746

[41] Sweeney M, Yu Y, Platoshyn O, Zhang S, McDaniel SS, Yuan JX. Inhibition of endogenous TRP1 decreases capacitative Ca2+ entry and attenuates pulmonary artery smooth muscle cell proliferation. Am J Physiol Lung Cell Mol Physiol 2002;283: L144–155. 10.1152/ajplung.00412.2001 12060571

[42] Wang C, Li JF, Zhao L, Liu J, Wan J, Wang YX, et al Inhibition of SOC/Ca2+/NFAT pathway is involved in the anti-proliferative effect of sildenafil on pulmonary artery smooth muscle cells. Respir Res 2009;10: 123 10.1186/1465-9921-10-123 20003325 PMC2797778

[43] Yang K, Lu W, Jia J, Zhang J, Zhao M, Wang S, et al Noggin inhibits hypoxia-induced proliferation by targeting store-operated calcium entry and transient receptor potential cation channels. Am J Physiol Cell Physiol 2015;308: C869–878. 10.1152/ajpcell.00349.2014 25740156 PMC4451349

[44] Wang Y, Lu W, Yang K, Wang Y, Zhang J, Jia J, et al Peroxisome proliferator-activated receptor gamma inhibits pulmonary hypertension targeting store-operated calcium entry. J Mol Med (Berl) 2015;93: 327–342. 25391250 10.1007/s00109-014-1216-4 PMC4334731

